# Regeneration of human bones in hip osteonecrosis and human cartilage in knee osteoarthritis with autologous adipose-tissue-derived stem cells: a case series

**DOI:** 10.1186/1752-1947-5-296

**Published:** 2011-07-07

**Authors:** Jaewoo Pak

**Affiliations:** 1 Miplant Stems Clinic, 32-3 Chungdam-Dong, Gangnam-Gu, Fourth Floor, Seoul, Korea

## Abstract

**Introduction:**

This is a series of clinical case reports demonstrating that a combination of percutaneously injected autologous adipose-tissue-derived stem cells, hyaluronic acid, platelet rich plasma and calcium chloride may be able to regenerate bones in human osteonecrosis, and with addition of a very low dose of dexamethasone, cartilage in human knee osteoarthritis.

**Case reports:**

Stem cells were obtained from adipose tissue of abdominal origin by digesting lipoaspirate tissue with collagenase. These stem cells, along with hyaluronic acid, platelet rich plasma and calcium chloride, were injected into the right hip of a 29-year-old Korean woman and a 47-year-old Korean man. They both had a history of right hip osteonecrosis of the femoral head. For cartilage regeneration, a 70-year-old Korean woman and a 79-year-old Korean woman, both with a long history of knee pain due to osteoarthritis, were injected with stem cells along with hyaluronic acid, platelet rich plasma, calcium chloride and a nanogram dose of dexamethasone. Pre-treatment and post-treatment MRI scans, physical therapy, and pain score data were then analyzed.

**Conclusions:**

The MRI data for all the patients in this series showed significant positive changes. Probable bone formation was clear in the patients with osteonecrosis, and cartilage regeneration in the patients with osteoarthritis. Along with MRI evidence, the measured physical therapy outcomes, subjective pain, and functional status all improved. Autologous mesenchymal stem cell injection, in conjunction with hyaluronic acid, platelet rich plasma and calcium chloride, is a promising minimally invasive therapy for osteonecrosis of femoral head and, with low-dose dexamethasone, for osteoarthritis of human knees.

## Background

Adipose-tissue-derived stem cells (ADSCs) have been widely used in Korea over the last few years by plastic surgeons as a semi-permanent volume expander. In June 2009, the Korean Food and Drug Administration (KFDA) allowed ADSCs to be used as autologous cell transplant when obtained and processed within a medical clinic with minimal processing [1].

Mesenchymal stem cells (MSCs) are found in numerous human tissues including bone marrow, synovial tissue and adipose tissue. These have been shown to differentiate into bones, cartilage, muscle and adipose tissue, representing a promising new area of therapy in regenerative medicine [2].

Because of their potent capabilities, MSCs have been used successfully in animal models to regenerate cartilage and bones [3,4]. In 2008, Centeno and colleagues reported regeneration of knee cartilage in a human by using autologous culture-expanded bone-marrow-derived stem cells [5]. However, to the best of our knowledge ADSCs have never been used successfully in osteonecrosis of a femoral head and in osteoarthritis of a human knee.

Osteonecrosis, or avascular necrosis, of femoral head is relatively a common disorder affecting individuals in their 30s to 50s. Osteoarthritis of a knee is an even more common disorder, especially in older patients. Currently, the only cure for both diseases is surgical intervention. However, the successful regeneration of bones and cartilage with ADSCs may represent a promising new, minimally invasive, non-surgical alternative.

Many issues need to be resolved and clarified before the general application of the procedure. The mechanism of regeneration is not yet clear. It could be through direct differentiation of stem cells that were introduced to the diseased joints. Alternatively, it could be due to the tropic effects of ADSCs on the existing tissues. Further, various elements of the local environment can affect the differentiation of MSCs [6]. Also, it is believed that a scaffolding material might be needed to allow the MSCs to attach and engraft [7].

Platelet-rich plasma (PRP) was used as a growth factor and as a differentiating agent for the MSCs. PRP contains multiple growth factors including transforming growth factor (TGF)β, insulin-like growth factor (IGF), fibroblast growth factor (FGF), and platelet-derived growth factor (PDGF). A literature review of the data on PRP shows that it has a positive effect on the stimulation of bones, blood vessel and chondrocyte formation [8-10]. Hyaluronic acid was added as a scaffolding material, and calcium chloride was used as a PRP-activating agent [11].

This series of case reports demonstrates successful clinical results of regenerating bones in osteonecrosis and cartilage in patients with osteoarthritis, using percutaneously implanted, autologous MSCs along with PRP, hyaluronic acid, calcium chloride (CaCl2) and very-low-dose dexamethasone.

## Case presentations

The following cases concern four different individuals. Of the four, the first two cases involve bone regeneration in osteonecrosis of hips, the latter two cases regeneration of cartilage in osteoarthritis of knees.

The first case concerns a 29-year-old Korean woman with over a year's history of right hip pain due to osteonecrosis.

Approximately a year prior to presentation, she started having hip pain with no history of trauma. She was seen by a physician and was diagnosed with osteoarthritis of the hip after an MRI scan. After taking non-steroidal anti-inflammatory drugs (NSAIDs) for a few weeks, her hip pain improved. About a month prior to presentation, she again started having hip pain radiating to the anterior region of the right knee. The pain was worse when standing up, walking, and exercising. The pain improved with rest. However, this time, the pain was not greatly relieved with NSAIDs.

A repeat MRI showed osteonecrosis of the femoral head, stage 4. Since there is no effective non-surgical treatment of the disease, she elected to receive stem cell treatment.

At the time of initial evaluation, she reported moderately severe pain (visual analog scale (VAS) score 7) on rest, increased pain when standing and walking (VAS score 9).

For a week prior to liposuction, she was restricted from taking corticosteroids, aspirin, NSAIDs, and oriental herb medications.

For the liposuction procedure, she was brought into an operating room and placed in a supine position. She was then sedated with propofol 2 mg intravenously (push) and a 20 mg/hour rate of continuous infusion.

After cleaning her abdominal area with povodine-iodine and placing sterile drapes, an incision of approximately 0.5 cm was made approximately 5 cm below the umbilicus. Then, using tumescent solution (500 cm^3 ^normal saline, 40 cm^3 ^2% lidocaine, 20 cm^3 ^0.5% marcaine, 0.5 cm^3 ^epinephrine 1:1000), the lower abdomen area was anesthetized. Next, using a 3.0 Hartman cannula, a total of 160 cm^3 ^of lipoaspirates were extracted and separated by gravity. The resulting 100 cm^3 ^of adipose tissue was then centrifuged at 3500 rpm for five minutes. The end result was approximately 40 cm^3 ^of packed adipose tissue, fibrous tissue, red blood cells and a small number of nucleated cells.

An equal volume of digestive enzyme, 0.07% collagenase type 1, composed of several collagenases, sulfhydryl protease, clostripain, a trypsin-like enzyme, and a amino peptidase, derived from *Clostridium histolyticum *(Adilase; Worthington, Lakewood, NJ, USA) was then mixed with the centrifuged lipoaspirates at a ratio of 1:1 and digested for 30 minutes at 37°C while rotating [12].

Bacterial collagenases differ from vertebrate collagenases in that they exhibit broader substrate specificity [13].

After the digestion, the lipoaspirates were centrifuged at 100*g *for three minutes to separate the lipoaspirate and the enzyme. The leftover enzyme was then removed.

Using 500 cm^3 ^5% dextrose in lactated Ringer's solution, the lipoaspirates were washed three times to remove the collagenase. After each washing, the lipoaspirates were centrifuged at 100 *g*. After the last centrifuge process, approximately 10 cm^3 ^of ADSCs were obtained.

While preparing the ADSCs, 30 cm^3 ^of autologous blood was drawn with 2.5 cm^3 ^of anticoagulant citrate dextrose solution (ACD) formula. This was centrifuged at 200 *g *for five minutes. The resultant supernatant was drawn and centrifuged at 1000 *g *for five minutes. The supernatant was drawn and discarded. The resulting buffy coat was mixed with 10 cm^3 ^of ADSCs.

Hyaluronic acid 1 cm^3 ^was added to this mixture to act as a scaffold. This PRP was again mixed with CaCl_2 _for activation of platelets at a ratio of 10:2 (PRP 10:2 CaCl_2_).

In order to inject the mixture of stem cells and PRP, our patient was first placed in a lateral position with her left side down. After cleaning with povodine-iodine and draping with sterile drapes, 2% lidocaine was used to anesthetize the hip at the femoral head region. Using a 22-gauge 3.5-inch needle, 17 cm^3 ^mixture of ADSCs, PRP, hyaluronic acid and CaCl_2 _were injected into the femoral head under ultrasound guidance.

She was then instructed to remain still with her leg elevated for 30 minutes to allow for cell attachment. On discharge home, she was instructed to maintain activity as tolerated. She returned to the clinic for four additional PRP injections with calcium chloride every week over a period of a month.

**Figure 1:**
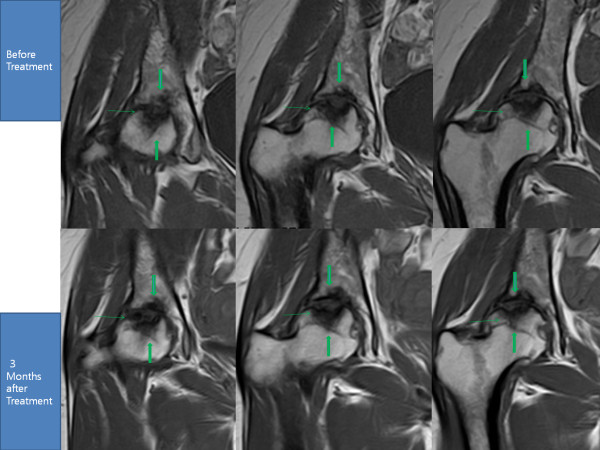
**MRI of the right hip; T1 sequential coronal views**. The cavity surrounded by the three green arrows has decreased in size in post-treatment MRIs due to probable bone regeneration.

After the fourth week of the ADSC injection, her pain had improved more than 50%. By week 12, her pain had improved more than 70% along with an improvement in range of motion (Tables 1 and 2). A repeat MRI taken at week 12 showed a significant filling of bone defects on the superior acetabulum and probable bone matrix formation in the subcortical region of the femoral head (Figure 1).

**Table 1:** Functional rating index [16] and visual analog scale (VAS) score for patient 1

Outcome measures	Pre-injection	four weeks post-treatment follow-up	12 weeks post-treatment follow-up
Functional rating index	15	12	8

VAS	7	4	2

**Table 2:** Physical therapy (PT) range of motion of patient 1

PT session	Flexion (degrees)	Flexion VAS	Abduction (degrees)	Abduction VAS	Adduction (degrees)	Adduction VAS
Pre-injection evaluation	91	5	20	6	10	7

four weeks post-treatment follow-up	110	3	35	3	15	3

12 weeks post-treatment follow-up	125	2	40	2	20	2

VAS = visual analog scale.

The second case concerns a 47-year-old Korean man who had been working as a diver until three years prior to presentation.

Approximately three years prior to presentation, he started having right hip pain and was diagnosed with osteonecrosis of the right hip. His pain had progressed over three years and he was offered a total hip replacement (THR). Being reluctant to proceed with the surgical procedure, he elected to proceed with stem cell treatment. Before the procedure, a repeat MRI of the hip was performed and a diagnosis of osteonecrosis of the femoral head, stage 4, was confirmed.

**Figure 2:**
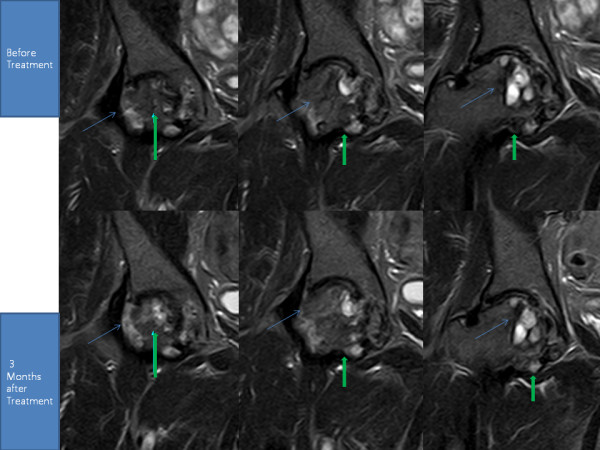
**MRI of the right hip; T1 sequential coronal views**. The blue arrow shows the pattern of probable bone regeneration. The green arrow shows probable bone consolidation.

He then underwent the same procedures as our first patient. After the fourth week of the ADSC injection, his pain improved more than 30% along with improvement in range of motion. However, by week 12, his pain had only minimally improved further (Tables 3 and 4). Interestingly, a repeat MRI taken at week 12 showed a significant filling of bone defects with a possibility of bone matrix formation at the site of necrosis in the femoral head (Figure 2).

**Table 3:** Functional rating index and visual analog scale (VAS) score of patient 2

Outcome measures	Pre-injection	four weeks post-treatment follow-up	12 weeks post-treatment follow-up
Functional rating index	16	12	12

VAS	8	5	5

**Table 4:** Physical therapy (PT) range of motion of patient 2

PT session	Flexion (degrees)	Flexion VAS	Abduction (degrees)	Abduction VAS	Adduction (degrees)	Adduction VAS
Pre-injection evaluation	90	8	15	8	10	8

four weeks post-treatment follow-up	100	5	20	5	10	5

12 weeks post-treatment follow-up	105	5	20	5	15	5

VAS = visual analog scale.

The third case concerns a 70-year-old Korean woman with over five years' history of right knee pain due to osteoarthritis.

Due to her occupation, she made active use of her bilateral knee joints. With a diagnosis of osteoarthritis of the right knee, she had received multiple injections of steroids and hyaluronic acid over the years. However, she did not notice any improvement in her pain. She was seen by an orthopedic surgeon and was offered a total knee replacement (TKR). She was reluctant to go through the TKR procedure due to possible side effects. Since then, she has been receiving physical therapy with little improvement.

At the time of initial evaluation, she reported moderately severe pain (VAS score 7) on rest. Her knee pain increased when walking. She also complained of mild knee swelling. On physical examination, there was mild joint swelling, a decreased range of motion and tenderness with flexion. Apley and McMurray tests were negative, and there was no ligament laxity.

A pre-treatment 1.5T MRI scan demonstrated a decreased size and deformed contour on the medial meniscus of the left knee due to maceration.

After obtaining ADSCs and preparing PRP as described above for the first two patients, she was prepared for injection of the mixture into the joint.

In order to inject the stem cell and PRP mixture, she was first placed in a supine position with her right knee bent at 90°. After cleaning with povodine-iodine and draping with sterile drapes, her knee was anesthetized with 2% lidocaine at the medial and lateral sides of the inferior patella. Using a 22-gauge 1-inch needle, 8.5 cm^3 ^of ADSCs, PRP, dexamethasone and hyaluronic acid mixture was injected into the medial and the lateral sides of the knee.

She was then instructed to remain still for 30 minutes to allow for cell attachment. As she was subsequently discharged from the clinic, she was instructed to maintain activity as tolerable.

**Figure 3:**
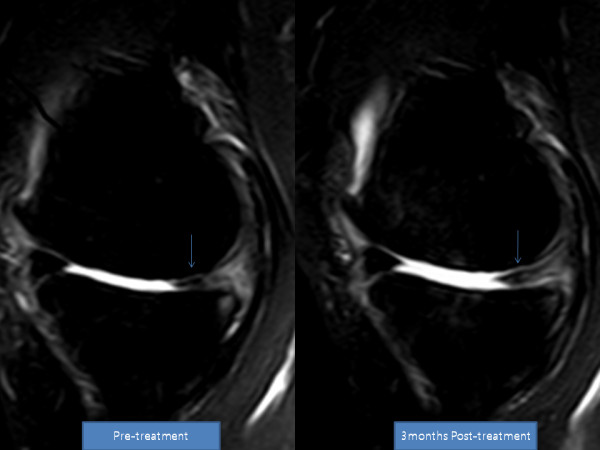
**MRI sagittal T2 view of the knee**. Pre-treatment and post-treatment MRI shows increased height of medial meniscus cartilage and articular cartilage (arrow).

**Figure 4:**
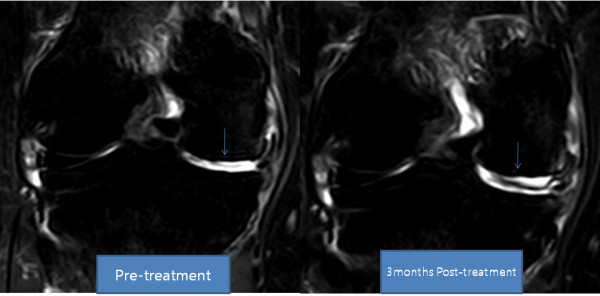
**MRI coronal T2 view of the knee**. Pre-treatment and post-treatment MRI shows increased height of medial meniscus (arrow).

She returned to our clinic for four additional PRP and dexamethasone injections over the next four weeks. After the seventh week of ADSC injection, her pain had improved more than 80% and flexion of the knee had also improved. By week 12, her pain had improved more than 90% and the range of motion also further improved (Tables 5 and 6). A post-treatment MRI taken at week 12 showed a significant increase in the thickness of meniscus cartilage on the medial side of the right knee (Figures 3 and 4).

**Table 5:** Functional rating index and visual analog scale (VAS) score of patient 3

Outcome measures	Pre-injection	seven weeks post-treatment follow-up	12 weeks post-treatment follow-up
Functional rating index	36	16	13

VAS	7	2	1

**Table 6:** Physical therapy (PT), range of motion of patient 3

PT session	VAS score	Flexion (degrees)	Flexion VAS	Extension (degrees)	Extension VAS
Pre-injection evaluation	7	110	7	+3	1/10

seven weeks post-treatment follow-up	2	130	3	+5	0/10

12 weeks post-treatment follow-up	1	130	2	+5	0/10

VAS = visual analog scale.

The final case concerns a 79-year-old Korean woman with over seven years' history of bilateral knee pain due to osteoarthritis.

Her left knee was much more painful than the right. Due to her occupation, she made active use of her bilateral knee joints. With a diagnosis of osteoarthritis of both knees, she had received multiple injections of steroids and hyaluronic acid in both knees over the years. However, she noticed no improvement of pain. She was seen by an orthopedic surgeon and was offered a TKR. She was also reluctant to go through the TKR due to possible side effects. Since then, she had been receiving physical therapy with little improvement.

At the time of initial evaluation, she reported severe pain in the left knee (VAS score 8) on rest. The pain was increased when walking. On physical examination, there was deformity of the knee, mild joint swelling, a decreased range of motion and tenderness with flexion. Apley and McMurray tests were negative, and there was no ligamentous laxity.

A pre-treatment 1.5T MRI demonstrated a decreased size and deformed contour on her medial meniscus of the left knee due to maceration. She also underwent the same procedure as our previous patient.

**Figure 5:**
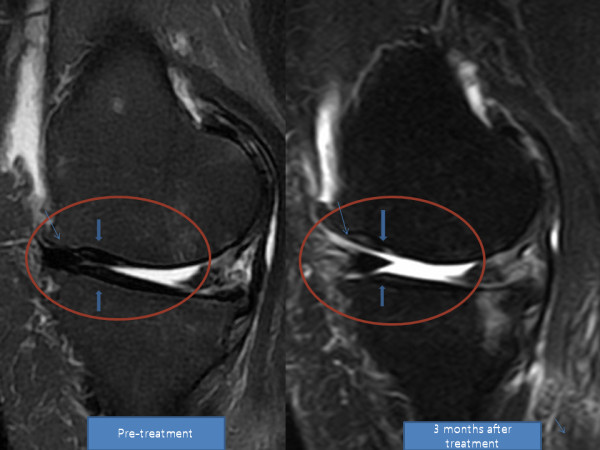
**MRI sagittal T2 view of the knee**. Pre-treatment and post-treatment MRI shows increased height of medial meniscus cartilage. The articular cartilage also has a clearer marking, representing probable cartilage regeneration.

**Figure 6:**
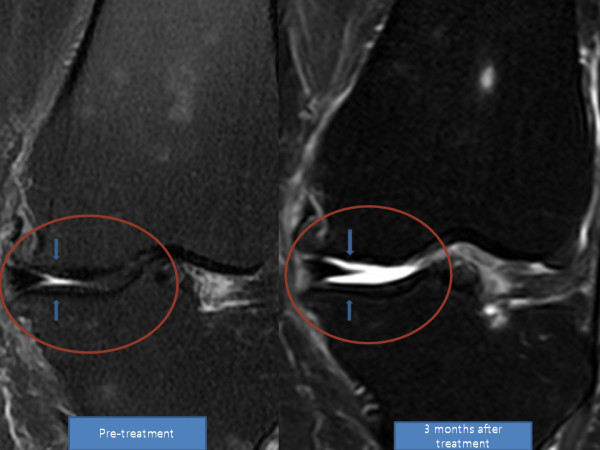
**MRI coronal T2 view of the knee**. The anterior medial meniscus has increased in height.

After the fourth week of ADSC injection, her pain improved over 50% and flexion of the knee improved as well. By week 12, her pain had improved over 90% and she was able to flex her knee further (Tables 7 and 8). A repeat MRI taken at week 12 showed a significant increase in the height of her meniscus cartilage on the anterior medial side of the left knee (Figures 5 and 6).

**Table 7:** Functional rating index and visual analog scale (VAS) score of patient 4

Outcome measures	Pre-injection	four weeks post-treatment follow-up	12 weeks post-treatment follow-up
Functional rating index	36	20	13

VAS	8	4	1

**Table 8:** Physical therapy (PT) range of motion of patient 4

PT session	VAS score	Flexion (degrees)	Flexion VAS	Extension (degrees)	Extension VAS
Pre-injection evaluation	8	110	7	+3	1/10

four weeks post-treatment follow-up	4	120	5	+4	0/10

12 weeks post-treatment follow-up	1	130	2	+5	0/10

VAS = visual analog scale.

## Discussion

This series of clinical case reports provides clear MRI evidence of apparent bone regeneration in osteonecrosis of femoral heads and meniscus cartilage regeneration in osteoarthritis of human knees. Based on the MRI features, it is probable that the new tissue formation is bone matrix in the case of osteonecrosis and meniscus cartilage in osteoarthritis. However, without biopsy, the true nature of the newly-formed tissue is unclear. While bone and cartilage regeneration using ADSCs has been shown in animal models, these case reports represent the first successful regeneration of bones and cartilage in human patients.

In addition to the MRI evidence, the patients' symptoms and signs also improved. It is worthwhile to note that the patients' symptoms improved gradually over three months. Thus, it can be speculated that, in patients with osteonecrosis, newly-formed bone has concomitant neovascularization. Osteonecrosis, or avascular necrosis, occurs due to compromise in blood circulation. Without concurrent neovascularization, the consolidation or regeneration of bones cannot be sustained.

Another issue with these clinical results is that patients with osteoarthritis did not report 100% symptom improvements. This may be due to the fact that osteoarthritis is a disease of the whole knee, not just the cartilage.

With regard to the mechanism of tissue regeneration, there are a few plausible possibilities. The mechanism of regeneration could be through direct differentiation of stem cells that were introduced through the injection. However, there is a possibility that the ADSCs exert tropic effects on the existing tissues as well. Numerous studies have reported that MSCs, in addition to tissue repair and regenerative effects, have immunomodulatory and paracrine effects [14].

Furthermore, PRP could have contributed to the regeneration of bones and blood vessels. PRP contains multiple growth factors including TGFβ, IGF, FGF, and PDGF. A literature review of the data on the uses of PRP showed that it has a positive effect on the stimulation of bones and blood vessels and chondrocytes. Here, it was used as a growth factor and as a differentiating agent for the MSCs.

Further, dexamethasone injection, used as a differentiating agent for cartilage, may also have had positive effects in patients with osteoarthritis. The levels injected (100 ng/mL) were negligible compared to the doses being used in clinical settings. Such low doses in the nanogram range have been shown to increase extracellular matrix production by chondrocytes, and are commonly used *in vitro *to differentiate MSC from cartilage [15].

This is the first series of case reports showing possible successful bone and cartilage regeneration in humans by using a combination of ADSCs, hyaluronic acid, PRP and CaCl_2_. Currently, no non-surgical therapy is available for the treatment of osteonecrosis and osteoarthritis. Thus, stem cell therapy may significantly improve current treatment strategies for the treatment of knee osteoarthritis and osteonecrosis of the femoral head. However, further studies need to be initiated to find out the true detailed nature of the apparently regenerated bones and cartilage and to determine the true mechanism of tissue regeneration.

## Conclusions

After three months of treatment, all the patients reported on above were able to straighten their hips and extend their knees further, affecting MRI postures. Therefore, obtaining the post-treatment MRI data at the exactly same location as pre-treatment MRI of the hips and knees was difficult.

Although there were difficulties in repeatedly obtaining the exact location of the hips and knees, the pre-procedure and post-procedure MRI analyses clearly demonstrate filled bone defects in osteonecrosis and increased meniscus cartilage volume in osteoarthritis, indicating regeneration attributable to the ADSC treatment. Additionally, the measured physical therapy outcomes, subjective pain, and functional status, all improved

## Consent

Written informed consent was obtained from all patients for publication of this case report and any accompanying images. Copies of the written consents are available for review by the Editor-in-Chief of this journal.

## Competing interests

The authors declare that they have no competing interests.

## Authors' contributions

JP was in charge of patient treatment and follow-up, was responsible for manuscript drafting and revision, and read and approved the final manuscript.
